# Production of High Flux Poly(Ether Sulfone) Membrane Using Silica Additive Extracted from Natural Resource

**DOI:** 10.3390/membranes10010017

**Published:** 2020-01-19

**Authors:** Sri Mulyati, Syawaliah Muchtar, Mukramah Yusuf, Nasrul Arahman, Sofyana Sofyana, Cut Meurah Rosnelly, Umi Fathanah, Ryosuke Takagi, Hideto Matsuyama, Norazanita Shamsuddin, Muhammad Roil Bilad

**Affiliations:** 1 Department of Chemical Engineering, Universitas Syiah Kuala, Jl. Syeh A. Rauf, No. 7. Darussalam, Banda Aceh 23111, Indonesia; sri.mulyati@unsyiah.ac.id (S.M.); syawaliah2009@gmail.com (S.M.); mukramah@mhs.unsyiah.ac.id (M.Y.); sofyana71@unsyiah.ac.id (S.S.); cut.meurah@che.unsyiah.ac.id (C.M.R.); umifathanah@unsyiah.ac.id (U.F.); 2 Graduate School of Environmental Management, Universitas Syiah Kuala, Jl. Tgk Chik Pante Kulu No. 5, Darussalam, Banda Aceh 23111, Indonesia; 3 Research Center for Environmental and Natural Resources, Universitas Syiah Kuala, Jl. Hamzah Fansuri, No. 4, Darussalam, Banda Aceh 23111, Indonesia; 4 Research Center for Membrane and Film Technology, Department of Chemical Science and Engineering, Kobe University, Rokkodai-Cho 1-1, Nadaku, Kobe 657-8501, Japan; takagi@harbor.kobe-u.ac.jp (R.T.); matuyama@kobe-u.ac.jp (H.M.); 5 Faculty of Integrated Technologies, Universiti Brunei Darussalam, Jalan Tungku Link, Gadong BE1410, Brunei; norazanita.shamsudin@ubd.edu.bn; 6 Chemical Engineering Department, Universiti Teknologi PETRONAS, Seri Iskandar, Perak 32610, Malaysia; mroil.bilad@utp.edu.my

**Keywords:** polyethersulfone, rice husk, bagasse ashes, membrane surface modification

## Abstract

This paper reports the application of silica derived from natural biomasses of rice husk and bagasse ashes as membrane modifying agents. The modification was conducted on poly(ether sulfone) (PES) membrane by blending the silica into the dope solution. The modification was aimed to improve the structure and hydraulic performance of the resulting PES membrane. The effects of silica addition to the membrane system were evaluated through the analysis of change in chemical structure using ATR-FTIR, surface morphological change using AFM, and surface hydrophilicity using water contact angle measurement. SEM and AFM images show the silica loading significantly affects the membranes morphologies. Silica loading also promotes hydrophilic property as shown by the decrease in water contact angles from 82° to 52–60° due to the presence of polar groups in some residual silica in the membrane matrix. Silica blending also leads to the formation of membranes with higher permeability of up to three folds but lower humic acid rejection (78–62%). The findings indicate the role of silica to enhance the membrane pore size. The ability of membrane to reject humic acid (of 0.8 nm minimum diameter) indicating that the resulting membranes were in between tight ultrafiltration and nanofiltration type. Nonetheless, applying too-high silica concentration decreased the humic acid rejection most likely due to over enlargement of the membrane pore size.

## 1. Introduction

Membrane technology has been widely applied in water and wastewater treatment processes. The membrane process is widely used for removal of particles, turbidity, organic substances, and microorganisms in water [1,2,3] as it offers many advantages including low energy consumption, easy operation, and produces high quality of water [4,5]. Polyethersulfone (PES) is one of the commonly used polymers in the fabrication of nanofiltration (NF), ultrafiltration (UF), and microfiltration (MF) membranes. This polymer has a high glass transition temperature (T_g_), excellent mechanical strength, outstanding stability against chemicals, and high resistance to oxidation processes [5,6]. However, PES is a hydrophobic with a low antifouling potential [7]. When PES membrane is applied to the filtration process, solute that cannot pass through the membrane can be easily adsorbed onto the surface of the hydrophobic polymer matrix and lowers membrane permeability and decreases membrane performance [8].

Surface modification is an effective way to improve the antifouling properties of membranes. Improving the surface hydrophilicity of the membrane is one of the most attractive methods to reduce the membrane fouling because it reduces the interaction between foulant and membrane surface, and hence, lowers the deposition of foulant on the membrane surface [9]. Many investigations have proven that increasing membrane surface hydrophilicity effectively reduces the fouling [10,11]. There are four general approaches to improve the hydrophilicity of PES membranes, namely, by (1) grafting, including photo-induced grafting and thermal-induced grafting, (2) surface coating, (3) plasma treatment, and (4) mixing PES polymers with hydrophilic materials [12,13]. 

Among the above modification methods, polymer blending with hydrophilic substances is the easiest because it can result in a membrane with a more stable and constant structure. Some hydrophilic additives have been used by several researchers for this purpose including hydrous manganese dioxide nanoparticles [14], polydopamine and polyethylene glycol [9], pluronic F127 [15], and PEG/Al_2_O_3_ [7]. Moreover, the addition of nanoparticles such as SiO_2_, Fe_3_O_4_, Al_2_O_3_, ZrO_2_, TiO_2_, and ZnO affect the resulting membrane characteristics, namely pore size, thickness, hydrophilicity and parameters related to membrane structures, such as porosity and formation of macrovoid [5]. Nano silica is a high-potential modifying material for membrane due to its ability to form OH bond and its low toxicity in water. In addition, it can increase the hydrophilicity of PES membranes and minimize fouling on the membrane surface [16]. Thus, silica is considered as an excellent membrane fabrication additive.

Not only chemical silica but also the silica derived from natural product (i.e., strobe silica) has been used to increase membrane hydrophilicity [5]. Rice husk and bagasse are abundant natural sources of silica [13], and can be used as sustainable source for the silica additive for membrane fabrication. In this study, SiO_2_ extracted from local rice husk and local sugarcane bagasse ashes sourced from Banda Aceh, Indonesia was employed as additives to improve the hydrophilicity and hydraulic performance of PES membranes. This study aims to investigate the effects of the blending of silica rice husk and sugarcane bagasse biomass, and its concentration on the characteristics, and the performance of the resulting PES membrane by using pristine PES membrane as reference. Characteristics of the prepared membranes were evaluated in terms of chemical composition, water contact angle using water contact angle meter, morphology, and pure water permeability. The membrane type was indirectly estimated by filtration of 10 ppm humic acid in water. 

## 2. Materials and Methods 

### 2.1. Materials

PES as the main polymer of membrane material was supplied from BASF Co. (Ludwigshafen, Germany). N-methyl Pirrolidone (NMP) purchased from Merck (Hohenbrun, Germany) was used as the solvent. Distilled water was used as the non-solvent. Aceh’s rice husk waste and sugarcane bagasse waste were used as the source to extract silica to be used as the additive. Humic acid (sodium salt, technical grade 50–60%) supplied from Sigma Aldrich (STL, MO, USA) was used as a model organic foulant.

### 2.2. Isolation of Silica from Rice Husk and Sugarcane Bagasse 

The preparation method of silica from the rice husk and the sugarcane bagasse biomasses has been explained in our previous work [14]. The extraction procedure using a solvent was carried out to isolate silica compounds. A total of 50 grams of rice husk or sugarcane powder were dissolved in KOH (37%) at a temperature of 30 °C. HCl (37%) was then added into the solution to precipitate the silica. The formed silica was then removed from the solution through filtration. The dry silica was further processed by a ball mill for 20 h to reduce the particle size. An X-ray fluorescence (XRF, PANalytical, Almelo, the Netherlands) was provided to confirm the purity of the isolated silica from rice husk and sugarcane bagasse. From the XRF analysis, it is found that the concentration of silica content in the rice husk and sugarcane bagasse were 93.4 and 85.6 (%), respectively. The full composition of silica particles used in this study is shown in Table 1.

### 2.3. Preparation of Membrane 

PES membrane was prepared by the phase inversion technique. Silica additives (from rice husk or sugarcane bagasse silica) with various concentrations of 0, 3, and 5 wt% were loaded into the PES in N-Methyl-2-pyrrolidone (NMP) solution, then stirred until it was completely homogeneous. The detail compositions of the casting solutions are shown in Table 2. Each of the homogeneous solution was then cast on a glass plate using a casting knife with a thickness of 300 µm. After casting, the glass plate was immediately immersed into a coagulation bath containing the non-solvent (distilled water) to allow phase separation to progress. The membrane formation was allowed until the membrane film was detached naturally from the glass plate. Following that, the membranes underwent annealing process. It was done by immersing the membrane in hot water at temperature of 70 °C for 10 minutes. The thickness of the M1, M2, M3, M4, and M5 were 0.08, 0.03, 0.04, 0.05, and 0.04 mm, respectively. The effect of membrane thickness on the filtration performance is not discussed in detail because of the asymmetric structure of the membrane, in which surface pore plays most prominent role.

### 2.4. Analysis of Membrane Morphology

The membrane surface was observed using the Scanning Electron Microscopy (SEM, JSM-7500F, JEOL Ltd., Tokyo, Japan). Prior to observation, the membrane was dried using a freeze dryer (Eyela, EDU-1200, Tokyo, Japan) for 24 h, then coated with gold. The membrane surface morphology was observed under 10 kV. SEM analysis was carried out to obtain qualitative characteristics of the membrane, both surface and macrovoid structures. For the SEM measurement, the analysis was carried out only for M1 (pure PES), M3 (PES + 5 wt% rice husk silica) and M5 (PES + 5 wt% sugarcane bagasse silica) membranes. The surface morphology of the membrane samples were further measured by Atomic Force Microscopy (AFM, SII NanoTechnology Inc., SPA400, Tokyo, Japan). The outer surface of the membrane was imaged in the scanning area of 1 µm × 1 µm. After five repetitions, the average roughness (Ra) was obtained using Spicel 32 software.

### 2.5. Water Contact Angle Measurement

Water contact angle of the membrane with and without modification with silica additives was measured using the Drop Master 300 (Kyowa Interface Science Co., Saitama, Japan). 0.5 mL of distilled water was dropped onto the membrane surface using the micro syringe. Water contact angle was measured approximately 10 times per membrane on different random locations and reported as the average value.

### 2.6. Fourier Transform Infrared Spectroscopy (FTIR)

To observe the changes in the membranes chemical structure before and after modification, the Attenuated Total Reflection Fourier Transform Infrared (ATR-FTIR) analysis was carried out using a FTIR-8100A Fourier Transform Infrared Spectrophotometer (Shimadzu Co., Kyoto, Japan). Prior to measurement, membrane samples were dried in a desiccator at a room temperature for 2 h. Following that, the sample was placed above the sample holder. Absorbance data was measured at wave numbers range of 400–4000 cm^−1^. Furthermore, an X-ray photoelectron spectroscopy (XPS; JPS-9010, JEOL, Tokyo, Japan) has been carried out to confirm the existence of silica in the M1, M3, and M5 membrane. The sample was kept in the sample chamber for twelve h at room temperature to release the moisture. The composition of chemicals on the surface membrane was determined with an Al Kα radiation source (1486.6 eV).

### 2.7. Filtration Performance

A laboratory-scale of dead-end filtration module was conducted to analyze the hydraulic performances of the membrane. One sheet film with an effective surface area of 0.003 m^2^ was fixed up at the bottom of the module. For flux investigation, deionized water was passed through the top membrane layer with transmembrane pressure of 3.0, 3.5, and 4.0 bar. The filtration process was run until the constant permeation rate was achieved. The flux of membrane was determined by using Equation (1):

(1) $$Flux\;\left(L/m^{2}h\right)=\frac{V}{A\cdot t}$$

where *V* is the volume of permeate (L), *A* the effective surface area of the membrane (m^2^), and *t* filtration time (h).

The coefficient of pure water permeability (*Lp*) is the ability of a membrane to pass pure water based on the operating pressure. The value of *Lp* is obtained from the slope of the flux as a function of the operating pressure.

The estimation of membrane pore size was done via solute rejection method by filtration of humic acid with a molecular weight of 226 Da according the data provided by the supplier. The 10 ppm humic acid solution was used as the feed. The feed was forced through the membrane using the dead-end filtration system under a trans-membrane pressure of 3.5 bar. The filtration was carried out for 2 h. Humic acid concentration before and after filtration was analyzed using a Spectrophotometer. The percentage of rejection was calculated using Equation (2):

(2) $$Rejection\;\left(\%\right)=\left(\frac{C_{f-\;}C_{p}}{C_{f}}\right)\;\times 100\%$$

where *C_f_* indicates the concentration of humic acid in the feed solution (ppm) and *C_p_* indicates the concentration of humic acid in the permeate (ppm).

## 3. Results and Discussion

### 3.1. Effects of Silica on Membrane Morphology

**Figure 1:**
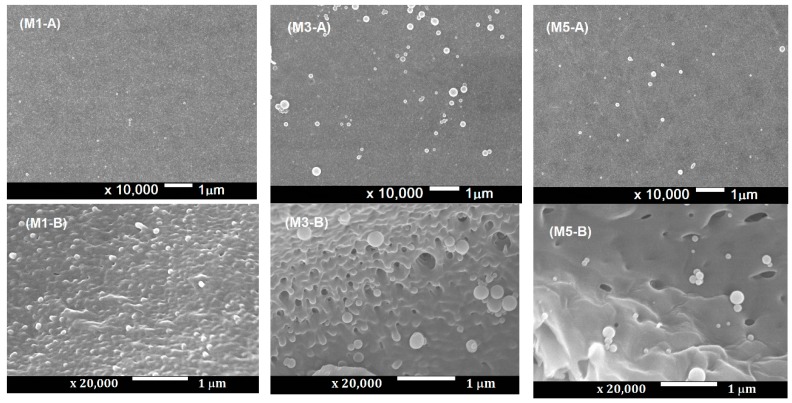
SEM imaging of the (**A**) surface and (**B**) macrovoid structure of the M1, M3, and M5 membranes.

The results of SEM analyses of M1, M3, and M5 membranes are shown in Figure 1. The imaging results showed that original PES membrane (M1-A) has a smooth surface, while the addition of silica from both biomass sources (M3-A, M5-A) caused the membrane to have a rougher surface. The ‘A’ legend (for example, M1-A) indicates the surface structure, and the ‘B’ legend indicates the magnified image, important for visualizing the macrovoid structure. The magnified images (M1-B, M3-B, M5-B) provide closer view of the pore structure of the membranes. Both silica from the rice husk or the sugarcane bagasse is expected to increase the pore size. This is due to the presence of hydrophilic silica nanoparticles in the membrane matrix, which attracts water and causing the nucleation and growth of water phase in the matrix of polymeric membrane. The hydrophilic property of the nano-silica enhances the exchange rate of solvent and non-solvent during the phase inversion, which then accelerates the de-mixing process. The growth of the polymer lean phase decreased with increasing viscosity of casting solution which led to the dispersion of small water droplets which formed as pores in the matrix of the membrane [14] indicated by the whit dots on the membrane surface (Figure 1).

**Figure 2:**
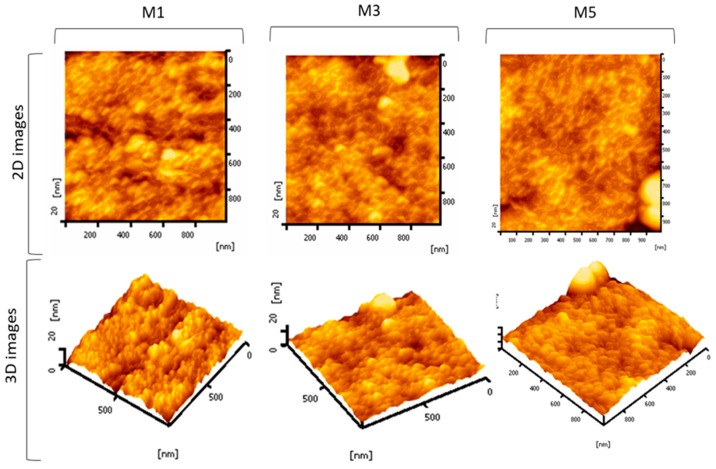
The two-dimensional (2D) and three-dimensional (3D) AFM images of the selected membrane samples.

To further observe the effect of the silica additive on membrane surface morphology, the surface roughness was measured by AFM [17]. Figure 2 shows the two-dimensional (2D) and the three-dimensional (3D) images of the membrane samples with and without silica blending. The bright areas represent peaks whereas the dark areas represent valleys corresponding to the membrane pores. Since the surface pore are is not clearly visible through the surface SEM image and no other pore size characterization was conducted, the effect of silica loading on the pore size of the membrane could not be demonstrated. However, as detailed later, it can be deduced from the clean water permeability and humic acid rejection data.

The roughness of pure PES membranes (M1), membranes modified with silica from rice husk M3 and sugarcane bagasse M5 of 1.966 nm, 4.56 nm, and 2.039 nm, respectively. The surface roughness of the membranes varies but with no obvious trend. The high surface roughness of M3 is caused by the nodule-like morphology which formed on the top of membrane surface as can be seen in Figure 2. The formation of such geometry can be attributed to adherence of the residual silica-polymer matrix formed as the polymer-lean phase.

### 3.2. Membrane Hydrophilicity

Contact angle is an effective indicator for membrane hydrophilicity [18]. Lower contact angle indicates higher hydrophilicity of the membrane [12]. Hydrophilic membrane surface plays an important role in improving the stability of permeation and the antifouling properties of the membrane [19]. Improvement of membrane hydrophilicity is very important if the hydrophobic polymer such as PES is used as a membrane material [7].

**Figure 3:**
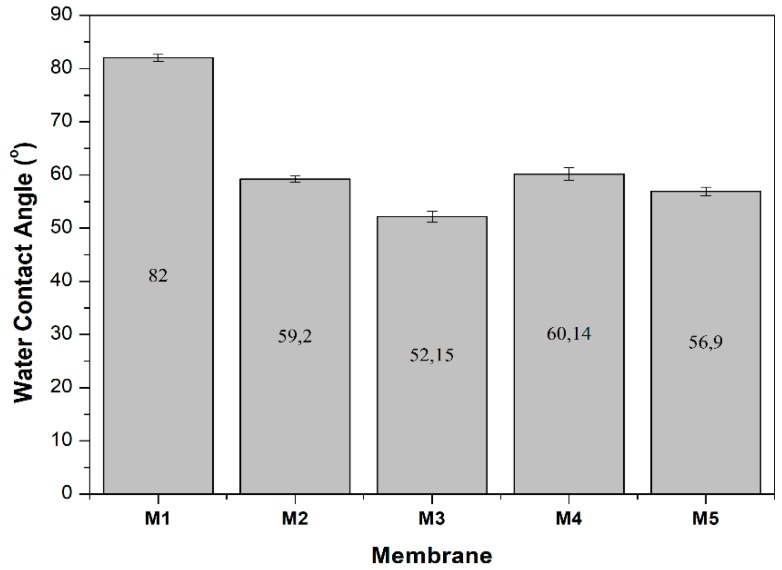
Water contact angle of prepared membranes.

Figure 3 shows the water contact angle of membranes demonstrating the efficacy of silica additive in improving membrane surface hydrophilicity. The hydrophilic property is attributed to the hydrophilic hydroxyl groups on the surface of the silica particles. This finding also indicates that not all silica particles leached out during the phase inversion. In addition, it is seen that the silica loading, irrespective of the sources, improves the hydrophilicity of the membrane as compared to the reference (M1). 

### 3.3. Membrane Chemical Composition Analysis

**Figure 4:**
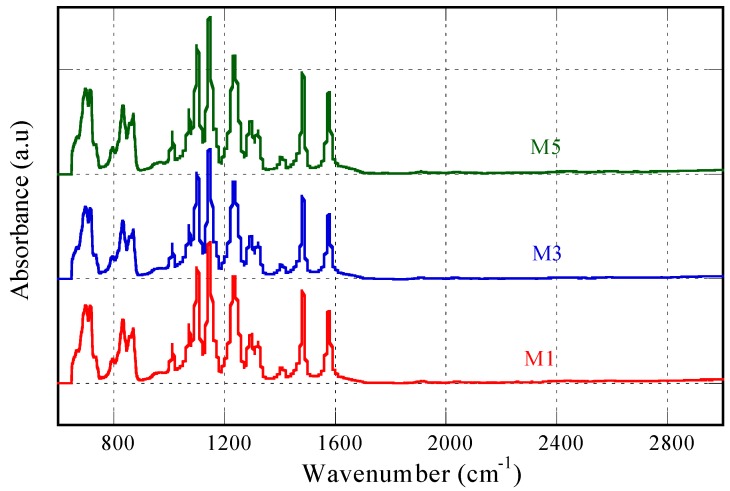
IR Spectrum of pure (M1) and silica modified PES membrane sourced from rice husk (M3) and sugarcane bagasse (M5).

The results of IR analysis for PES membranes with and without silica addition are shown in Figure 4. ATR-FTIR analysis was used to determine the chemical structure or functional group of polymers. No significant change in the peaks location as well as intensity was detected on the PES membrane without (M1) and with silica addition (M3 and M5). For all the membrane samples, strong bands at wavenumber of 1577 cm^−1^ and 1460 cm^−1^ are detected which assigned to the aromatic bands [20]. The big absorbance peak appeared in the range of 1200–1000 cm^−1^ typifies the stretching vibration of C–O–C from aliphatic ether bond. From this result, it can be concluded that no physicochemical interaction occurred between PES and the silica additive. No sign of silica was detected in the IR spectra of blended membranes. The stability of the particle on the membrane matrix was not tested and beyond the scope of the study. Our current focus is toward the characteristic of the resulting membranes by employing bio-based silica as additive. However, substantial leaching of silica particle is expected during the phase inversion, a common phenomenon [21,22] due to hydrophilic nature of the silica particle that has stronger affinity to water than to the polymer matrix. Nonetheless, some fraction of silica can be entrapped, which is enough to induce surface hydrophilicity as later proven by contact angle measurement. 

The FTIR analysis reveals the properties of near the surface and a more thorough analysis is required on the full thickness of the membranes. The X-ray photoelectron spectroscopy (XPS) analysis was performed to detect the C, O, S, and Si elements for the M-series membranes. It was found that the M1, M3, and M5 contained 0.0%, 4.3%, and 0.9% of Si elements. The absence of Si in the M1 is as expected since no silica loading was performed on M1 dope solution. The presence of Si detected by XPS data but not by FTIR suggests the complete leaching of Si near the membrane surface. However, higher content of Si in the M3 over M5 is not in agreement with the applied loadings in their respective dope solutions indicating different degree of leaching. Silica leaching is more profound for the M5. The residual silica in the membrane matrix may further leach out during the membrane filtration but was not investigated in this study.

### 3.4. Filtration Performance

**Figure 5:**
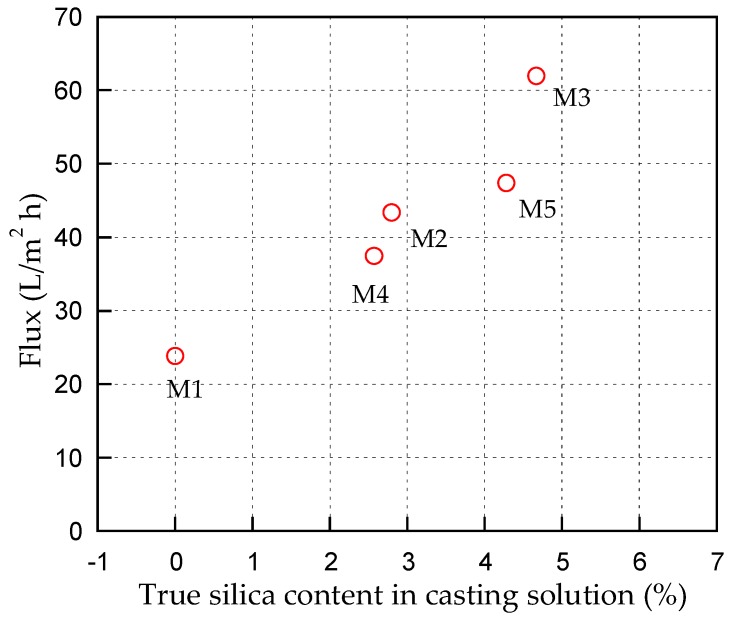
Effect of concentration and type of biosilica on pure water flux obtained at trans-membrane pressure of 3.5 bar.

The effect of silica types and their concentration on pure water flux is shown in Figure 5. The permeability data reflect important information on the intrinsic membrane properties and a key factor for practical membrane applications. Pure water permeability also reflects morphology of the membrane such as pore size, pore size distribution, and so on [23]. It is seen that the pure water flux of M1 (pure PES) membrane was only 23.8 L/(m^2^h). The pure water flux increased with increasing silica loading in the casting solution irrespective of the silica sources. This is because the silica is a hydrophilic additive. According to Arthanareeswaran et al. [24], hydrophilic property of a membrane increased pure water flux as it can reduce hydraulic resistance and increase membrane pore size and porosity. The addition of rice husk silica in casting solution generated higher water flux than that of with addition of silica from sugarcane bagasse. It is presumably due to higher content of silica produced from the rice husk ash compared to the sugarcane bagasse ash.

Table 3 shows membrane permeability coefficient (*Lp*) of all membranes. *Lp* is an intrinsic nature of membrane, which is indirectly used as an indicator to determine the hydraulic resistance and the membrane porosity [24]. In addition, membrane permeability is an essential characteristic that is a key specification for classifying membranes [25]. Pure PES membrane (M1) has the lowest *Lp* of 10.3 L/(m^2^h bar). It is found from Table 3 that the addition of silica and increasing its loadings increase the *Lp* of the membrane. For example, the *Lp* raises from 10.3 L/(m^2^h bar) to 18.8 L/(m^2^h bar) after the addition of 3 wt% rice husk silica (M2), and increases further to 29.5 L/(m^2^h bar) when the concentration increases to 5 wt% (M3). However, blending 3% and 5 wt% of sugarcane bagasse silica increases *Lp* from 10.3 L/(m^2^h bar) to 16.779 and 23.7 L/(m^2^h bar), respectively. The increase in the *Lp* of silica-modified membranes can be contributed by the improved hydrophilicity of the membrane [26]. Membranes modified with rice husks silica showed a higher *Lp* than that of sugarcane bagasse silica. This is because the content of SiO_2_ from the rice husk is higher than in the sugarcane bagasse as detailed in Table 1. 

**Figure 6:**
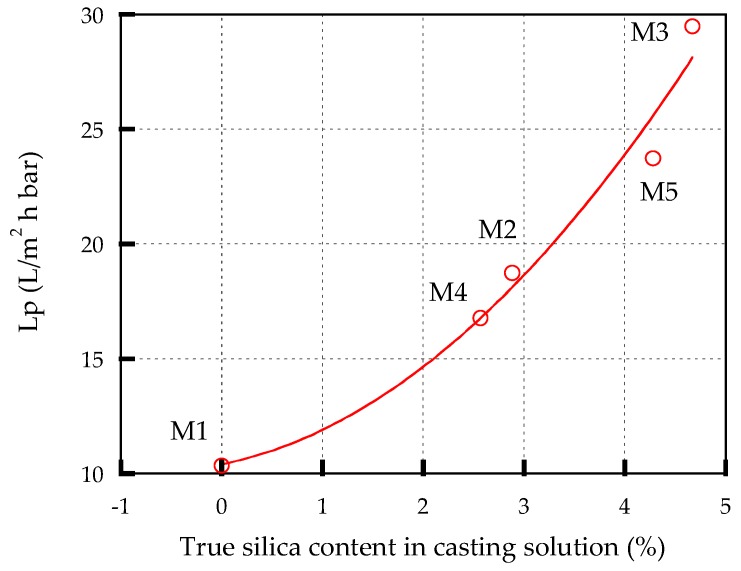
*Lp* as a function of true silica content in casting solution.

Figure 6 shows the *Lp* as a function of the true silica content (%) of casting solution. The true silica content (%) was obtained by (silica content (%) of casting solution × silica content of biomassa), for example, for M2, 3 × 0.934 = 2.802 (%). It is found from Figure 6 that *Lp* increases with the increase of true silica content of casting solution. Thus, it is clear the characteristic properties of the membrane depend on the silica content, and the impurities of biomass do not affect the performance of modified membrane.

Pore size measurement from surface SEM image is largely affected by the image resolution and magnification. The pore size of the membrane samples in this study could not be detected by the capillary flow porometer because for pressure limit of maximum 200 bar. And application of solute rejection method for molecular cut-off determining was difficult because of the close value of the estimated membrane pore sizes, judging from the clean water permeability trend. After carefully looking into the humic acid rejection data we concluded that the membrane is in between tight UF and NF range. The conclusion was made by estimating the minimum size of the humic acid according to a method detailed elsewhere [27], which could be very well rejected by all membrane samples (with humic acid rejection of > 62%). The applied humic acid had a molecular weight of 226 Da, corresponding to a minimum diameter of 0.8 nm, which fall under NF or tight UF classification [28], see Table 3.

**Figure 7:**
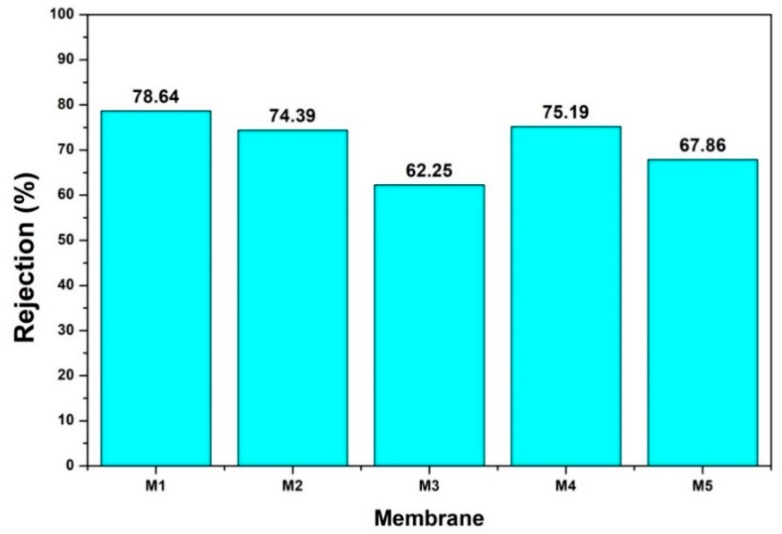
Humic Acid (10 ppm) rejection of PES membranes without and with addition of silica from rice husk and sugarcane bagasse of different concentrations. Filtrations were conducted at transmembrane pressure of 3.5 bar.

Figure 7 shows the humic acid rejection of pure and silica blended PES membranes. Separation degree (rejection) is an important parameter to evaluate the performance of membrane [29]. The feed solution, which contained 10 ppm humic acid was streamed over the prepared membranes using a filtration module. The results revealed that rejection of M1 membrane was 78.64%. Meanwhile, after modified with silica from the rice husk (M2, M3) and the sugarcane bagasse (M4, M5), the rejection declined gradually with increasing of silica loadings. 

The *Lp* and humic acid rejection data can be used to indirectly predict the pore properties of the membrane. High humic acid rejection of M1 indicating the pore size of slightly above humic acid molecular size of 0.8 nm. Upon loading of silica additive, the increase in the clean water permeability coupled by the decrease in the humic acid rejection upon higher loading of silica on the dope solution indicating the role of silica in enhancing pore size (which lead to both higher clean water permeability and lower humic acid rejection). This finding suggests the pore-forming property of silica, which increase the pore size of the blended membranes. Therefore, the use of silica additive from renewable sources is highly recommended for fabrication of PES-based NF/UF membranes.

## 4. Conclusions

Modification of PES membranes using silica obtained from biomass wastes of rice husk and sugarcane bagasse has been successfully conducted. Silica from both natural sources was added to the dope solution by means of blending, and the membranes were prepared through the non-induced phase separation technique. Results show the addition of silica significantly affects the resulting membranes morphologies, as evidenced by the results of SEM and AFM. In addition, silica additive promotes hydrophilic property as evidenced by the decrease in water contact angles from 82° to 52–60°. Simultaneously, silica blending also leads to production of membranes with higher permeability of up to three folds increment but lead to lower humic acid permeability from 78 to 62%. The finding suggests that silica loading enlarge the pore size resulting in higher clean water permeability and lower humic acid rejection. Overall, the use of silica additive from renewable sources is highly recommended for fabrication of PES-based NF/UF membranes.

## Figures and Tables

**Table 1:** Composition of silica particles.

Composition	Silica Particle from Rice Husk (%)	Silica Particle from Sugarcane Bagasse Biomasses (%)
SiO_2_	93.4	85.6
Al_2_O_3_	3.1	1.98
CaO	1.0	4.4
K_2_O	0.7	5.2
MgO	0.65	0.4
Fe_2_O_3_	0.45	2.2
NaO	0.22	-
Loss on ingnition	0.48	0.22

**Table 2:** Composition of casting solution.

Membrane	PES (wt%)	Silica (wt%)			NMP (wt%)
		Rice Husk	Bagasse	True Silica	
M1	17.5	0	0	0	82.5
M2	14.5	3	0	2.88	82.5
M3	12.5	5	0	4.67	82.5
M4	14.5	0	3	2.57	82.5
M5	12.5	0	5	4.28	82.5

**Table 3:** Membrane permeability coefficient (*Lp*).

Membrane	*Lp* (L/m^2^·h·bar)	Type of Membrane
M1	10.346	NF/UF
M2	18.753	NF/UF
M3	29.480	NF/UF
M4	16.779	NF/UF
M5	23.740	NF/UF
